# Genome-wide identification of the *HSF* gene family in Chinese chestnut and functional characterization of *CmHSF4* under temperature stress

**DOI:** 10.3389/fpls.2026.1749489

**Published:** 2026-03-06

**Authors:** XiuRong Xu, ZiQi Wu, Xibing Jiang, Shiming Cheng

**Affiliations:** 1 Zhejiang Academy of Forestry, Hangzhou, Zhejiang, China; 2 Zhengzhou University, Henan, China; 3 Research Institute of Subtropical Forestry, Chinese Academy of Forestry, Hangzhou, China

**Keywords:** Chinese chestnut, gene family, genome-wide identification, HSF, temperature stress

## Abstract

Heat shock transcription factors (HSFs) are key regulatory factors involved in plant responses to abiotic stress. To explore the *HSF* gene family characteristics in the Chinese chestnut (*Castanea mollissima*) (*Cm*) and its role in abiotic stress responses, we conducted a systematic analysis at the whole-genome level. Using bioinformatic methods, 18 *CmHSF* genes in the Chinese chestnut genome were identified, and these were unevenly distributed across 12 chromosomes. Phylogenetic analysis classified them into three subfamilies, A, B, and C, with the largest number of members (12) in A. Protein-conserved motif and gene structure analyses indicated that the internal structures of each subfamily were conserved, whereas significant differences existed among subfamilies, suggesting functional differentiation. Synteny analysis revealed that segmental duplication was the main driving force for the expansion of this family and that the genes were under strong purifying selection. *Cis*-acting element analysis showed that the promoter regions of *CmHSF* genes were rich in elements related to abiotic stress (such as hypoxia and low temperature) and hormonal responses, suggesting their involvement in complex stress regulatory networks. Expression pattern analysis further revealed the diversity of *CmHSF* gene functions. The transcriptome data indicated that different members exhibited complex and specific expression patterns under shade, low temperature, high temperature, and drought stress. Among them, the *CmHSF4* gene was particularly prominent under high-temperature stress, and its expression level increased sharply by 324 times at 4 h, indicating that it is a core candidate gene for understanding Chinese chestnut responses to heat stress. Subcellular localization experiments confirmed that *CmHSF4* is localized in the nucleus, which is consistent with its characteristics as a transcription factor. Preliminary functional verification in transgenic tobacco showed that high-temperature stress induced significant oxidative damage (increased MDA and H_2_O_2_ content), laying the foundation for further in-depth research on the stress resistance function of *CmHSF4*.

## 1 Introduction

Chinese chestnut (*Castanea mollissima* Blume) is an important woody tree species in China. The fruit of the tree (Chinese chestnuts) are rich in nutrients and have significant economic and ecological value. However, Chinese chestnuts are often threatened by various abiotic stresses during their growth and development, such as high temperature, drought, and salinity. Of these stresses, extreme high-temperature weather caused by global warming is becoming increasingly frequent and severe, and it is one of the key environmental factors restricting the improvement of Chinese chestnut yield and quality. To breed superior stress-resistant Chinese chestnut varieties through the use of molecular breeding methods and ensuring the sustainable development of the industry, it is therefore necessary to determine stress-resistant genetic resources and identify the key stress-resistance genes.

Adverse environmental stresses such as cold, high temperature, drought, and salt can severely affect seed germination, growth, development, yield, and plant quality (Kowalczewski et al., 2020). Heat-shock transcription factors (HSFs) play a core regulatory role in the complex molecular networks of plant responses to high-temperature stress. When plants are exposed to heat stress, denatured proteins accumulate in the cytoplasm, activating HSFs, and causing them to form trimers and translocate to the nucleus. HSFs contain five special structures: the N-terminal DNA-binding domain, oligomerization domain, nuclear localization signal, C-terminal transcriptional activation domain, AHA, and nuclear export signal, which are also known as HSF domains. The first *HSF* gene in plants was cloned from tomato (*Lycopersicon esculentum*) (Scharf et al., 1990). In the nucleus, HSF specifically recognizes and binds to heat shock elements (HSE, with the conserved sequence 5’-AGAAnnTTCT-3’) in the promoters of downstream target genes through its highly conserved N-terminal DNA-binding domain (DBD) (Jiang et al., 2021). This initiates the transcription of numerous stress response genes, including heat shock proteins, which jointly assist in the repair of damaged proteins and maintaining intracellular homeostasis, thus establishing heat tolerance (Baniwal et al., 2004).

Typical plant HSF proteins have a modular structure, including a DBD, an HR-A/B region responsible for protein oligomerization (Oligomerization Domain, OD), a nuclear localization signal, a nuclear export signal, and a C-terminal transcriptional activation domain, where the short peptide motif specific to class A HSF is called the AHA motif (Guo et al., 2008). Based on the structural characteristics of the HR-A/B region, plant HSFs are classified into three major classes: A, B, and C. Of these, class A is generally regarded as the main positive regulator of the high-temperature response. The AHA motif is a specific transcriptional activation motif in HSFA, whereas HSFB and HSFC lack transcriptional activation activity because of the absence of the AHA motif (Scharf et al., 1990). The heat stress elements (HSEs) of HSFs are formed by the repetitive pattern of the palindromic binding motif upstream of the HS-induced gene TATA box, and they usually require two or more HSE motifs (Nover et al., 2001).

Many reports of functional studies on members of the *HSF* gene family have been published. For example, the HSF family in *Camellia sinensis* comprises 22 members. Of these, both *CsHsf15* and *CsHsf16* have high induction rates under light and low-temperature stress, and contain *cis*-acting elements related to light and low-temperature responses, which indicates that they may play a role in light resistance and low-temperature stress (Li et al., 2025). Seventeen members have been identified in the carnation (*Dianthus caryophyllus*), and each member is upregulated by high temperatures and drought. Among them, four members, *DCAhSCF-A2A*, *DCAhSCF-A5*, *DCAhSCF-B2B*, and *DCAhSCF-C1* are slightly upregulated at low temperatures, which indicates that *DcaHsf* plays a key role in different stress response pathways (Li et al., 2019). Twenty-five HSF members have been identified in the Rosaceae fruit tree *Malus domestica*. At high temperatures, 12 were significantly higher than the reference sample, whereas only *MdHsfA9b* and *Mdhsfb4A-b* were strongly downregulated in response to an increase in temperature (Giorno et al., 2012). Therefore, members of the *HSF* gene family respond to various stressors through the activation or inhibition of plant growth. Many studies have confirmed that HSF proteins play important roles in plant responses to high-temperature stress. For example, the silencing of *HSFA1a* in tomatoes reduces the synthesis of chaperone proteins and *HsfA1a* proteins induced by heat stress, thereby increasing the sensitivity of *HSFA1A*-silenced tomato plants to heat stress. Furthermore, compared to wild-type plants, *HSFA2* mutant plants of *Arabidopsis thaliana* are more sensitive to heat stress at 37°C. In addition to heat stress, HSFs are involved in plant growth and other abiotic stresses (high temperature, low temperature, salt, and drought). For example, *HSFA9* is involved in the embryonic development and seed maturation of *Arabidopsis thaliana* and sunflower (Almoguera et al., 2002), and the four *HSF* genes (*HSFA1e*, *HSFA3*, *HSFA4a*, *HSFB2a*) of *Arabidopsis thaliana* are strongly induced by salt, low temperature and osmotic stress (Kilian et al., 2007).

With the extensive development of plant genome sequencing, the *HSF* gene family has been systematically identified at the whole-genome level in various plants, such as rice (*Oryza sativa* L.) (Baniwal et al., 2004), *Arabidopsis* (Guo et al., 2008), maize (*Zea mays*) (Lin et al., 2011), apple (*Malus domestica*) (Giorno et al., 2012), soybean (*Glycine max*), and cucumber (*Cucumis sativus* L.) (Chung et al., 2013). These studies have shown that the number of HSF family members varies significantly among different species and that they play diverse roles in response to high temperature and other abiotic stresses (such as low temperature, drought, and salt), presenting the coexistence of sub-functionalization and functional redundancy in the HSF family. These studies have provided an important foundation for understanding the roles and biological functions of the *HSF* gene family in the molecular mechanisms of high-temperature stress responses.

Based on this, this study employed bioinformatics methods to conduct the first genome-wide identification of the *HSF* gene family in Chinese chestnuts based on whole-genome data of Chinese chestnut. We systematically analyzed the physicochemical properties, evolutionary relationships, gene structures, and conserved motifs of the *HSF* gene family, and used transcriptome data to explore the expression patterns under various abiotic stresses. The results of this study provide a theoretical basis for understanding the biological functions of *HSF* genes in Chinese chestnuts and clarifying the molecular mechanisms of heat tolerance in Chinese chestnuts, with the aim offering guidance for obtaining valuable genetic resources to improve stress resistance in Chinese chestnuts and other forest crops.

## 2 Materials and methods

### 2.1 Identification, chromosome mapping and protein physicochemical property analysis of the *Castanea mollissima HSF* gene family

The preliminary data preparation involved downloading the genome data file and annotation file of the ‘N11-1’ chestnut variety from the Chinese National Genome Database (https://ngdc.cncb.ac.cn/gwh), the HSF domain file with the identifier PF00447 from the Pfam database (http://pfam-legacy.xfam.org/), and all 21 AtHSF protein sequences from the TAIR database (https://www.arabidopsis.org/) (Wang et al., 2021). Subsequently, the HSF domain and all AtHSF sequences were compared using the Simple HMM Search and Blast Several Sequences in the large database program of the TBtools software (Su et al., 2023), and the intersection of the comparison results of the two programs was uploaded to the InterProScan database (https://www.ebi.ac.uk/interpro/result/interprosca/). Finally, all members of the *CmHSF* gene family were identified (Chen et al., 2023).

After identifying all members, chromosome mapping and drawing of chromosome mapping diagrams for each *CmHSF* gene family member were conducted using the Gene Location Visualization (Advanced) program in TBtools software. They were named according to their chromosomal distributions.

To better understand the physicochemical properties of these CmHSF members, the amino acid quantity, molecular weight, isoelectric point, instability coefficient, and other indicators of CmHSF gene family member proteins were analyzed using the ExPASy ProtParam online tool (https://web.expasy.org/protparam/). The specific location of each family member protein in the cell, where it plays a specific role, was predicted using the BUSCA online tool (https://busca.biocomp.unibo.it/).

### 2.2 Construction of the evolutionary tree of *CmHSF* gene family

To better understand the grouping of the family members, we constructed phylogenetic trees for AtHSF and CmHSF. We first aligned all protein sequences using the MAFFT v7.471 program and employed MEGA7 software to construct a phylogenetic tree, which is displayed on the Chiplot website (https://www.chiplot.online/) (Xie et al., 2023). CmHSF was grouped based on the grouping of 21 AtHSF protein sequences in the TAIR database (https://www.arabidopsis.org/) and the phylogenetic tree.

### 2.3 Motif analysis and gene structure analysis of *CmHSF* gene family

The Simple MEME Wrapper program of TBtools software was used to analyze the motifs in the CmHSF protein sequences. Ten conserved motif sequences were obtained and uploaded to the InterProScan database (https://www.ebi.ac.uk/interpro/result/interprosca/) for functional annotation. Finally, the Gene Structure View of the TBtools software was used to visualize the motifs and gene structures (Chen et al., 2023; Xu et al., 2026).

### 2.4 Analysis of cis-regulatory elements in *CmHSF* gene family

Using the Fasta Extract program of the TBtools software, the 2000 bp promoter sequences preceding the ATG start codon of all *CmHSF* genes were extracted and uploaded to the PlantCARE database (http://bioinformatics.psb.ugent.be/webtools/plantcare/) for cis-regulatory element prediction. The number of such elements was counted, and the final results were presented as a heatmap using the HeatMap program in TBtools (Chen et al., 2023; Wang et al., 2012; Qiao et al., 2019).

### 2.5 Genomic collinearity analysis of *CmHSF* gene family

The collinearity of *CmHSF* gene family members within the Chinese chestnut genome was analyzed using MCScanX software, and a collinearity map was created using the Advanced Circos program in TBtools. The non-synonymous substitution rate/synonymous substitution rate (Ka/Ks) of the collinear gene pairs was calculated using the Simple Ka/Ks Calculator (NG) in TBtools. The replication types of the *CmHSF* gene family were analyzed using the DupGenfinder program and classified into four categories: whole-genome duplication (WGD), transposed duplication (TRD), dispersed duplication (DSD), and tandem duplication (TD) (Chen et al., 2023). The proximal duplication (PD) pattern was not observed in the *CmHSF* gene family members.

### 2.6 Genome-wide co-linearity analysis of *CmHSF* gene family

The genome and gff annotation files of Chinese chestnut, grape, and corn were downloaded from the National Genome Database of China (https://ngdc.cncb.ac.cn/gwh), and the genome and gff annotation files of *Arabidopsis thaliana* and rice were downloaded from the Ensembl Plants database (https://plants.ensembl.org/). The genome and annotation files of *Castanopsis tibetana* were downloaded from the CNGB database (https://db.cngb.org/); the genome and gff annotation files of Japanese chestnut were downloaded from the PlantGenomePortal database (https://plantgarden.jp/en/index); and the genome and gff annotation files of American chestnut were downloaded from the Phytozome13 database (https://phytozome-next.jgi.doe.gov/info/cdentata_v1_1.). MCScanX software was used to conduct a collinearity analysis between the Chinese chestnut genome and those of these species, and to determine the number of collinearity gene pairs. The formation time of each species was analyzed using the Timetree database (https://timetree.org/) to infer the time point at which the members of the *CmHSF* gene family were generated.

### 2.7 Expression patterns of *CmHSF* gene family members under abiotic stresses

The transcriptome FASTQ file with the accession number GSA: CRA022911 was obtained from the Chinese National Genome Database (https://ngdc.cncb.ac.cn/gsa), and the transcriptome was analyzed to explore transcriptome data of Yanbao under low light stress (0%, 50%, 75%, and 90%, which represents the gene expression status of two-year-old Yanbao chestnut seedlings after 10 d under shading intensities of 0%, 50%, 75%, and 90%, respectively).

The transcriptome FASTQ file with the accession number PRJNA1166987 was obtained from the NCBI database (https://www.ncbi.nlm.nih.gov/). The transcriptome was analyzed to explore the gene expression status of Yanshan Zao Feng chestnuts under low-temperature (D0h, D5h, D10h, and D15h) and high-temperature (G0h, G4h, G8h, and G12h) stress. D0h, D5h, D10h, and D15h represent the transcriptome data of Chinese chestnut seedlings at -15°C after 0, 5, 10, and 15 h, and G0h, G4h, G8h, and G12h represent the transcriptome data of Chinese chestnut seedlings at 45°C after 0, 4, 8, and 12 h.

The transcriptome FASTQ file (accession number: PRJNA731244) was obtained from the NCBI for Biotechnology Information database (https://www.ncbi.nlm.nih.gov/). The transcriptome was analyzed to explore the gene expression status of Yanshan Zao Feng chestnuts under drought (0d, 10d, 20d, 30d, 40d) stress, where 0d, 10d, 20d, 30d, and 40d represent the transcriptome data of Chinese chestnut seedlings under continuous water-free conditions for 0, 10, 20, 30, and 40 days.

All transcriptome data were sequenced using the Illumina platform in the paired-end mode. After obtaining the reads, they were aligned to the reference genome of Chinese chestnut N11-1, and the fragments per kilobase of transcript per million fragments mapped (FPKM) was used as the gene expression level.

### 2.8 Verification of relative expression level of *CmHSF4* under high-temperature stress using RT-qPCR

Leaves of one-year-old Chinese chestnut seedlings grown in a 45°C high-temperature stress environment for 0 h (G0h), 4 h (G4h), 8 h (G8h), and 12 h (G12h) were rapidly collected, frozen with liquid nitrogen, and stored at -80°C for subsequent gene cloning and qRT-PCR experiments. RNA was extracted from the tender leaves using the Plant RNA Extraction Kit (Takara, Beijing, China), and RNA purity was determined using a NanoPhotometer^®^ spectrophotometer (IMPLEN, CA, USA). RNA was reverse-transcribed into cDNA using the PrimeScriptTM RT Reagent Kit with gDNA Eraser (Takara, Beijing, China). RT-qPCR was performed on 12 samples (G0h, G4h, G8h, and G12h, four periods, with three replicates for each treatment, totaling 12 samples) using a SYBR PrimeScript RT-PCR Kit (Takara, Beijing, China). An ABI 7500 Real-Time PCR system (ABI 7500; Thermo Fisher Scientific, Singapore) was employed. The relative expression levels of the genes were calculated using the 2-ΔΔCT method. Fluorescent quantitative PCR primers were designed using the Batch qPCR Primer Design of the TBtools software. Primer information is provided in **Supplementary Table 1**, where CmActin is the internal reference gene for Chinese chestnuts. Bar graphs were plotted using GraphPad Prism (version 10).

### 2.9 Subcellular localization and tobacco transgenic verification

Using the seamless cloning technique, the ORF sequence of *CmHSF4* with the stop codon removed was ligated into the pAN580 vector, and a GFP tag was attached to the N-terminus. These recombinant vectors were then introduced into *Arabidopsis thaliana* protoplasts using polyethylene glycol-mediated transformation. Under 470 nm excitation, the GFP fluorescence signal was observed using a laser confocal microscope to analyze the localization of CmHSF4 in the cells.

Using the seamless cloning method, the ORF sequence of CmHSF4 was ligated into the pBWA(V)HS vector containing the CaMV35S promoter, and the recombinant plasmid was transformed into the root-knot bacterium strain GV3101. Transgenic tobacco plants were obtained by Agrobacterium infection of the tobacco leaves. Wild-type (WT) tobacco and overexpression (OE) tobacco were incubated at 45°C for 5 min, and the differences between WT tobacco and OE tobacco under high and normal temperatures were observed and photographed. The determination of H_2_O_2_ and MDA contents using the kits provided by Wuhan ProNets Biotechnology Co., Ltd (Wuhan, China).At the same time, spectrophotometers were used to measure the MDA (malondialdehyde) and H_2_O_2_ (hydrogen peroxide) values of WT tobacco and OE tobacco under high and normal temperatures (Kaseb et al., 2021; Deng et al., 2025).

Data analyses were performed using Excel and SPSS v21.0(International Business Machines Corporation, Amonk, New York, USA). Statistical significance was determined by the difference levels using a paired Student’s t-test. The mean standard deviation (SD) from the mean of at least three biological replicates are presented.

## 3 Results

### 3.1 Chromosome localization and protein physicochemical properties analysis of the *CmHSF* gene family

After searching the Chinese chestnut genome data and comparing them using MAGA7.0, 18 *CmHSF* genes were identified, and these were named CmHSF1–CmHSF18, based on the positions of the genes on the chromosomes (**Table 1**). The physicochemical properties were predicted. The sequence analysis results showed that the protein sequences encoded by CmHSFs varied greatly, ranging from 204 (CmHSF5) to 515 (CmHSF18) amino acids, with corresponding molecular weights of 23.64–56.97 kD, and theoretical isoelectric points (pI) of 4.63 (CmHSF2) to 8.08 (CmHSF3). The instability index ranged from 31.56 (CmHSF12) to 66.16 (CmHSF15), with an average value of 54.18. Protein hydrophobicity analysis revealed that the average hydrophobicity ranged from -0.899 (CmHSF10) to -0.455 (CmHSF1), indicating that all CmHSFs were hydrophilic proteins, but there were certain differences in hydrophilicity. The most and least hydrophilic were CmHSF10 and CmHSF1, respectively, but most CmHSFs were both unstable and hydrophilic. Prediction of the subcellular localization of the proteins showed that all CmHSFs were located in the nucleus.

**Table 1:** Basic characteristics of the putative proteins encoded by *CmHSF*s.

Gene ID	Chromosome ID	Gene name	Number of amino acid	Molecular weight	Theoretical pI	Instability index	Aliphatic index	Grand average of hydropathicity	Subcellular localization
EVM0015595	CmHSF1	Chr1	334	37271.16	5.59	55.30	68.29	-0.455	nucleus
EVM0029317	CmHSF2	Chr1	461	52774.3	4.63	66.15	80.69	-0.644	nucleus
EVM0031998	CmHSF3	Chr1	362	40750.02	8.08	47.90	69.20	-0.561	nucleus
EVM0015246	CmHSF4	Chr1	386	43240.12	4.80	51.93	75.26	-0.562	nucleus
EVM0025110	CmHSF5	Chr2	204	23648.91	7.60	54.47	69.36	-0.68	nucleus
EVM0007654	CmHSF6	Chr2	392	44801.05	5.08	55.47	71.33	-0.696	nucleus
EVM0007790	CmHSF7	Chr4	389	45139.93	4.71	51.30	69.90	-0.748	nucleus
EVM0018836	CmHSF8	Chr6	275	32032.17	6.95	43.27	70.51	-0.645	nucleus
EVM0028816	CmHSF9	Chr7	365	41898.06	5.39	56.39	69.40	-0.768	nucleus
EVM0010861	CmHSF10	Chr7	357	41531.42	5.32	58.72	65.21	-0.899	nucleus
EVM0018972	CmHSF11	Chr7	488	54821.75	5.53	64.29	58.18	-0.849	nucleus
EVM0022912	CmHSF12	Chr7	303	32867.23	5.54	31.56	61.75	-0.78	nucleus
EVM0003816	CmHSF13	Chr9	321	36026.15	5.74	53.43	69.88	-0.725	nucleus
EVM0013238	CmHSF14	Chr9	473	52210.06	4.81	46.04	75.48	-0.525	nucleus
EVM0008927	CmHSF15	Chr10	515	57846.48	4.73	66.16	69.81	-0.639	nucleus
EVM0019959	CmHSF16	Chr12	362	41218.54	5.56	58.85	63.26	-0.794	nucleus
EVM0007153	CmHSF17	Chr12	374	40517.68	4.73	51.32	69.79	-0.645	nucleus
EVM0015459	CmHSF18	Chr12	515	56971.41	5.02	62.70	64.16	-0.701	nucleus

**Figure 1:**
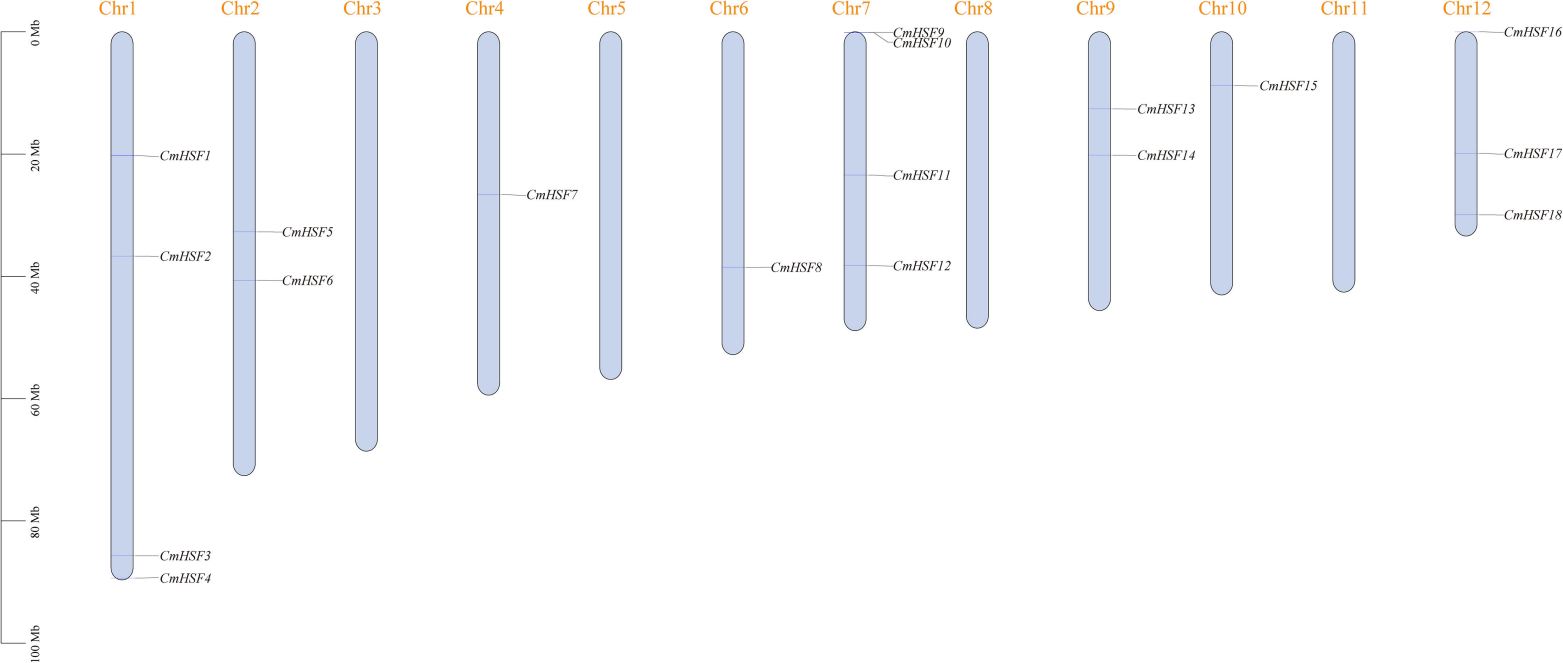
The istribution of the *CmHSF* genes on the Chinese chestnut chromosomes. Vertical colored bars represent the chromosomes of Chinese chestnut. The gene name and number are shown at the right/left of each chromosome. The scale bar on the left represents the length of the chromosomes.

Chromosome localization analysis revealed that the 18 *CmHSF* genes were unevenly distributed on the 12 Chinese chestnut chromosomes (**Figure 1**). Among them, members on chromosomes 1 and 7 were the most abundant, with four members each, followed by chromosome 12, with three members each; chromosomes 2 and 9, with two members each; and chromosomes 4, 6, and 10, with only one *CmHSF* gene each. No *CmHSF* genes were detected on chromosomes 3, 5, 8, or 11.

### 3.2 Phylogenetic analysis of *CmHSF* gene family proteins in Chinese chestnut and *Arabidopsis*

**Figure 2:**
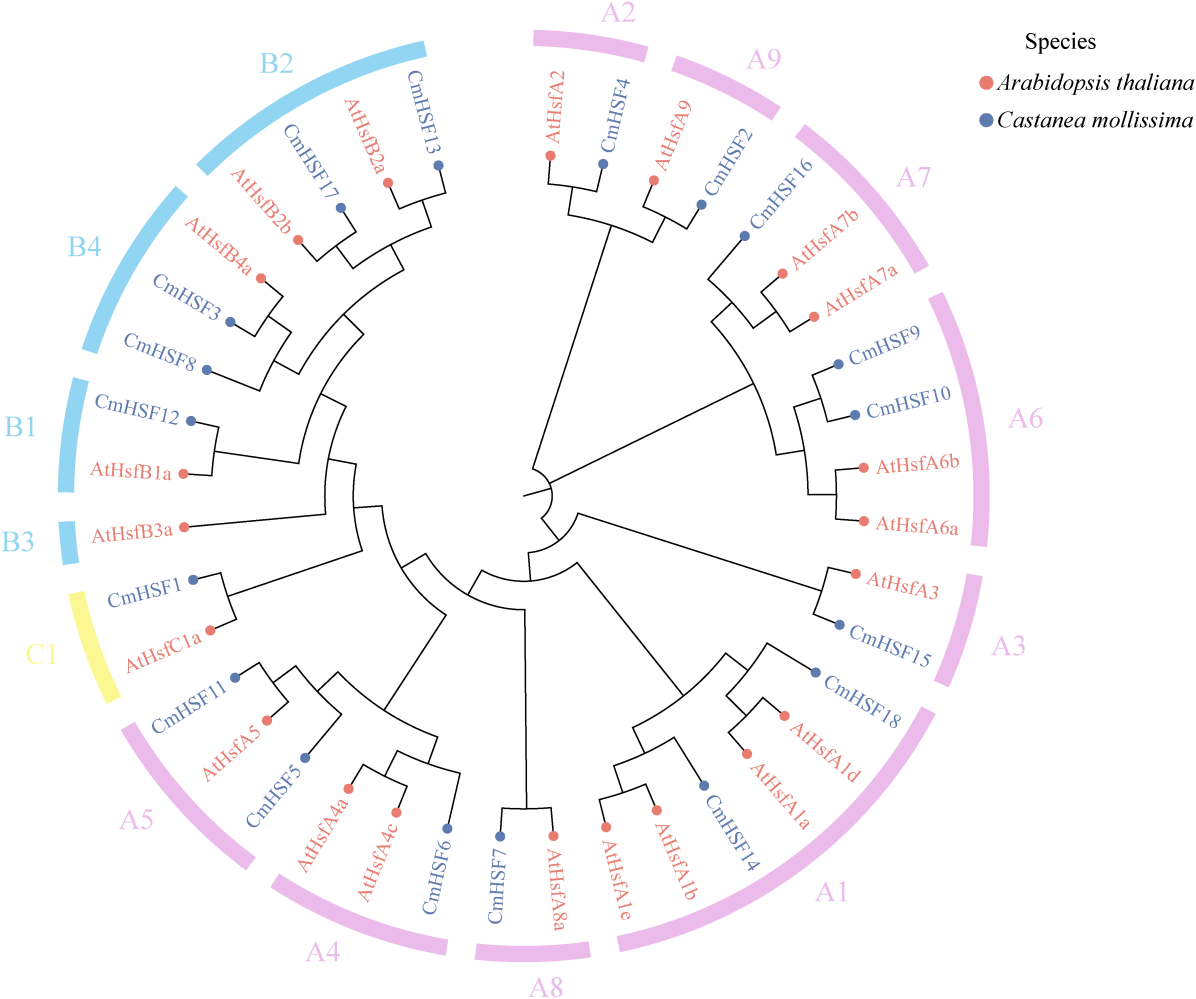
Phylogenetic tree based on the amino acid sequences of different species. Different branch colors in the figure indicate different groups; different colors of gene names indicate different orthogroups.

Phylogenetic analysis of the HSF family members in Chinese chestnut and *Arabidopsis* was conducted using MEGA software, and the results are shown in **Figure 2**. Based on the topological structure of the phylogenetic tree, the *HSF* gene families in Chinese chestnut and *Arabidopsis* were divided into three subfamilies (Class A, Class B, and Class C). Class C contained the fewest number of genes, with only one *CmHSF1* gene; Class B contained five *CmHSF* genes, namely *CmHSF3*, *CmHSF8*, *CmHSF12*, CmHSF*13*, and *CmHSF17*; and Class A contained the largest number of members, with 12 *CmHSF* genes, accounting for 70.59% of the total. The distribution of the number of genes in Chinese chestnut was similar to that observed in *Arabidopsis*. In addition, multiple *CmHSFs* were clustered with *Arabidopsis AtHSF* genes in the same branch, including *CmHSF13* in Class B with ATHsfB2a, *CmHSF17* with ATHsfB2b, and *CmHSF12* with ATHsfB1a. This indicated a high degree of relatedness and similar evolutionary trends, suggesting that they may have similar biological functions.

### 3.3 Prediction of CmHSF protein domain in chinese chestnut and analysis of gene structure

**Figure 3:**
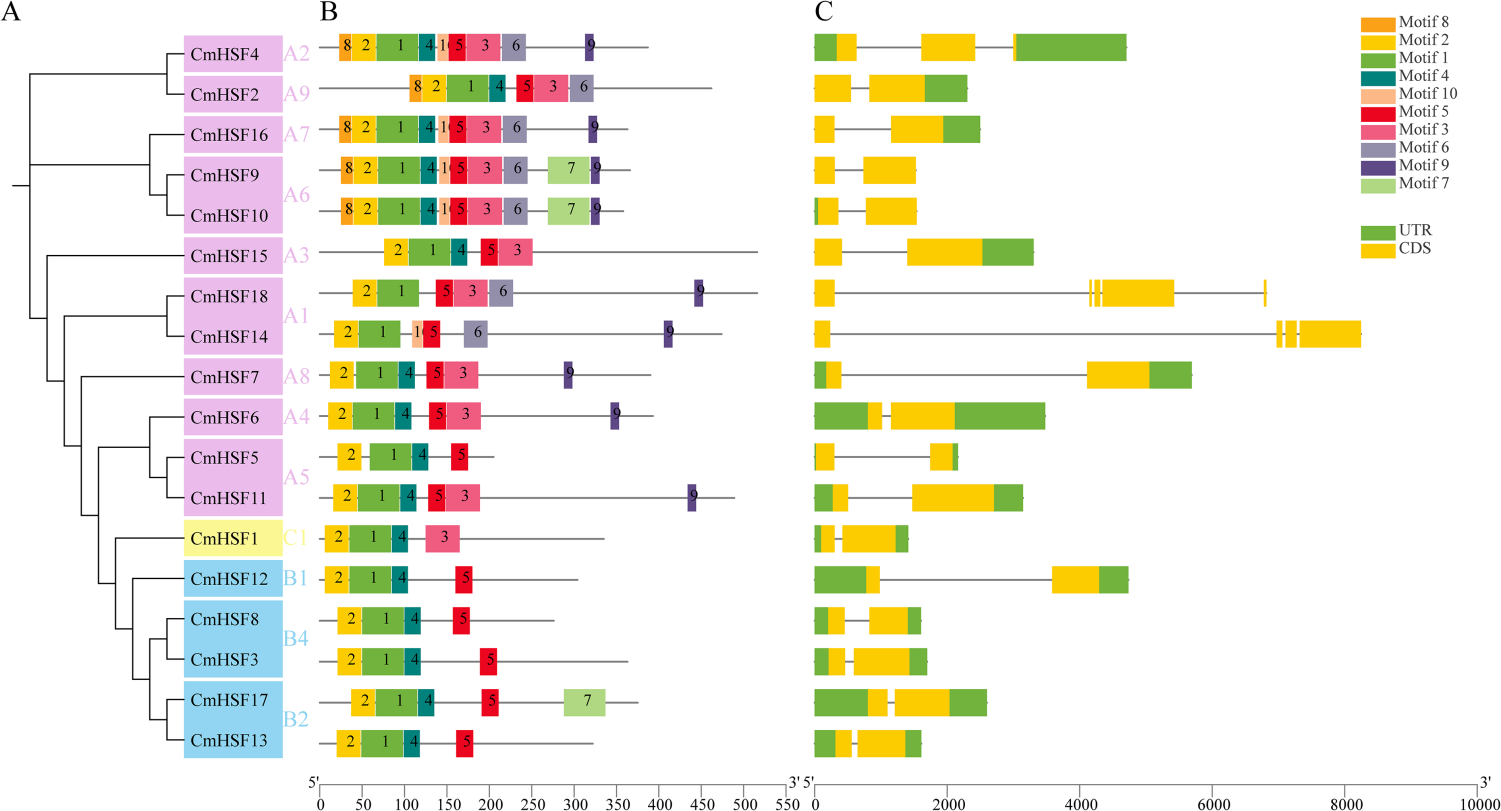
**(A)** The ten conserved motifs in CmHSFs. Conserved motifs of the CmDofs were identified using the online MEME program based on 25 full-length amino acid sequences with the following parameters: maximum number of motifs, 10; maximum width, 100. The lengths and positions of different motifs in the protein sequences are identified by the lengths and positions of the different color blocks. **(B)** Gene structure of *CmHSF*s. Exons, introns, and untranslated regions (UTRs) are indicated by yellow rectangles, black lines, and green rectangles, respectively.

The Chinese chestnut *CmHSF* domain was predicted using TB tools. The results showed that the number of motifs in Chinese chestnut *CmHSFs* ranged from four to ten. Among these, the Class C subgroup had the fewest motifs (four). Except for *CmHSF17*, which contained five motifs, all other members of the Class C subgroup contained only four motifs. Notably, all the *CmHSFs* contained motifs 1 and 2, which indicated that motifs 1 and 2 are relatively conserved *in CmHSFs* and may play important roles. Additionally, motifs 6–10 only appeared in the Class A subgroup. The motif structures of *CmHSFs* in the same evolutionary branch were similar, suggesting that *CmHSF* proteins in the same branch have similar functions. The different numbers and distributions of motifs among the family members suggest that different *CmHSFs* may have different biological functions (**Figures 3A, B**). Analysis of the *CmHSF* gene structure using TBtools (**Figure 3C**) revealed that the number of introns in *CmHSFs* ranged from one to four, and the number of exons ranged from two to five. Among these, *CmHSF*14 and *CmHSF*18 in the Class A subgroup contained the most introns. The most closely related *CmHSF* genes contained the same number of introns and intron and exon lengths, and the gene structures were similar.

### 3.4 Correlation analysis of *CmHSF* genes in Chinese chestnut

**Figure 4:**
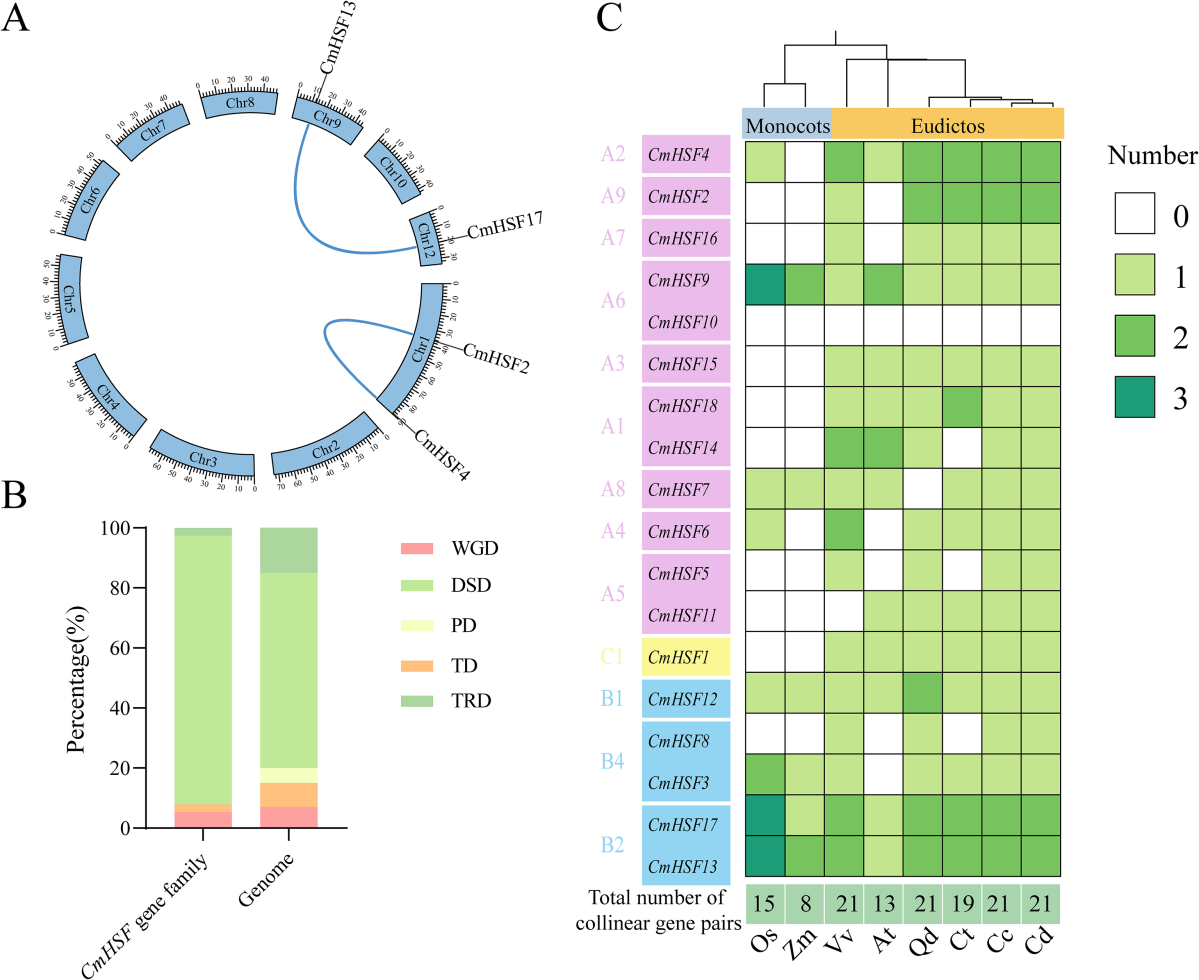
Analysis of collinearity and duplication types in the *CmHSF*gene family. **(A)** Intra-genomic collinearity of the *CmHSF*genes. **(B)** Duplication types of the *CmHSF*genes. TRD (Tandem Repeat Duplication), DSD (Dispersed Duplication), PD (Proximal Duplication), TD (Transposed Duplication) and WGD (Whole Genome Duplication) indicate transposon duplication, dispersed duplication, proximal duplication, tandem duplication and whole genome duplication in the figure. The same below **(C)** The number of genetic combinations of Chinese chestnut with other species.

To elucidate the potential evolutionary mechanisms of the Chinese chestnut *CmHSF* gene family, we studied the replication of these genes. The results (**Figure 4A**) indicated that among the 18 *CmHSF* genes, two fragments were duplicated, located on three different chromosomes, and two homologous gene pairs were formed. These paired correlated genes may have similar functions. Based on the correlation results, the analysis of gene replication types revealed that, compared to the entire Chinese chestnut genome, the PD replication type was completely lost in the *CmHSF* gene family, the proportions of TD and TRD were decreased, and the proportion of the DSD replication type were significantly increased (**Figure 4B**). Further Ka/Ks calculations were conducted, and the results showed that the Ka/Ks values of the two gene pairs were all less than 0.3 (**Supplementary Table S1**). Further correlation analyses were performed between Chinese chestnuts and other species, and a heat map was drawn. A further analysis of the correlation between Chinese chestnut *CmHSF* genes and those of *Arabidopsis thaliana*, rice, grape, and corn (**Figure 4C**) showed that there were different numbers of correlated gene pairs between Chinese chestnut and the different species. The number of gene pairs between Chinese chestnuts and corn was the lowest, with only eight genes. The number of gene pairs between Chinese chestnuts and grapes was the highest at 21. The heatmap also showed that the majority of Class A members were only correlated with dicotyledonous plants, suggesting that these Chinese chestnut *CmHSF* members formed after the differentiation of monocot and dicotyledonous plants. The genes *CmHSF12*, *CmHSF13*, and *CmHSF17* were correlated in all species, suggesting that they may play an important role in plant adaptation.

### 3.5 Analysis of promoter anterior regulatory elements of *CmHSF* gene family in Chinese chestnut

**Figure 5:**
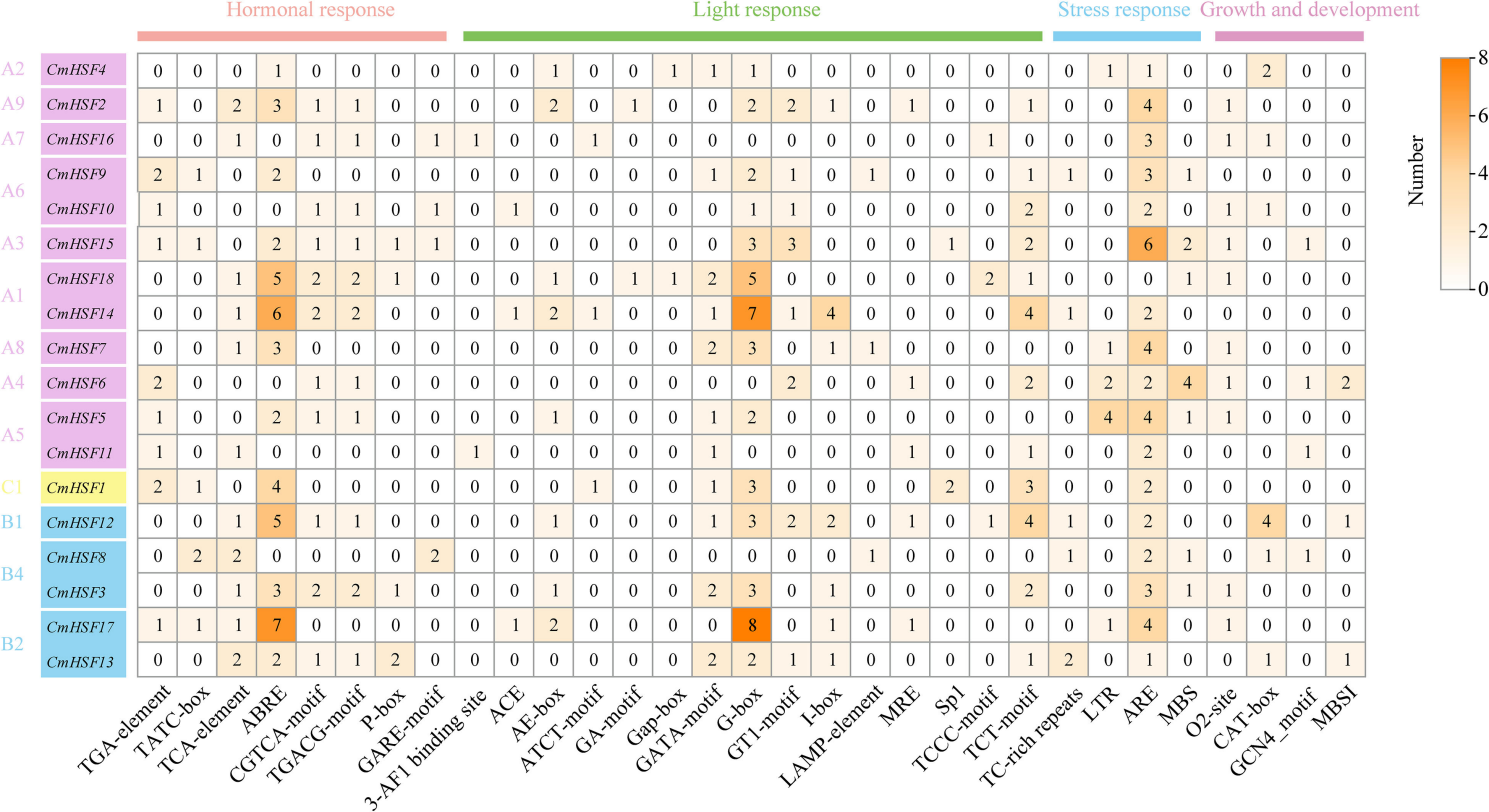
*Cis-* elements in the promoter regions of *CmHSFs.* The colored block with a number represents the *cis*-element number of *CmHSFs*.

The upstream promoter cis-regulatory elements, located at the beginning of the gene, serve as a reference for the biological function of the gene. This study analyzed the *cis*-regulatory elements of the 2,000 bp upstream promoter of *CmHSF*. As shown in **Figure 5**, the number of elements involved in biological and non-biological stress responses was the highest, including the cold response element (LTR) and anaerobic response element (ARE). Among them, ARE contained the highest number, which were present in almost all *CmHSF*s. The other elements were related to hormone responses, including (but not limited to) jasmonic acid methyl response elements (CGTCA-motif, TGACG motif) and salicylic acid response elements (TCA-element). The results of cis-regulatory element prediction further indicate that *CmHSFs* may have multiple biological functions and may be induced and regulated by hormones in response to non-biological stress.

### 3.6 Expression pattern analysis of *CmHSF* genes in different stress conditions of Chinese chestnut plants

**Figure 6:**
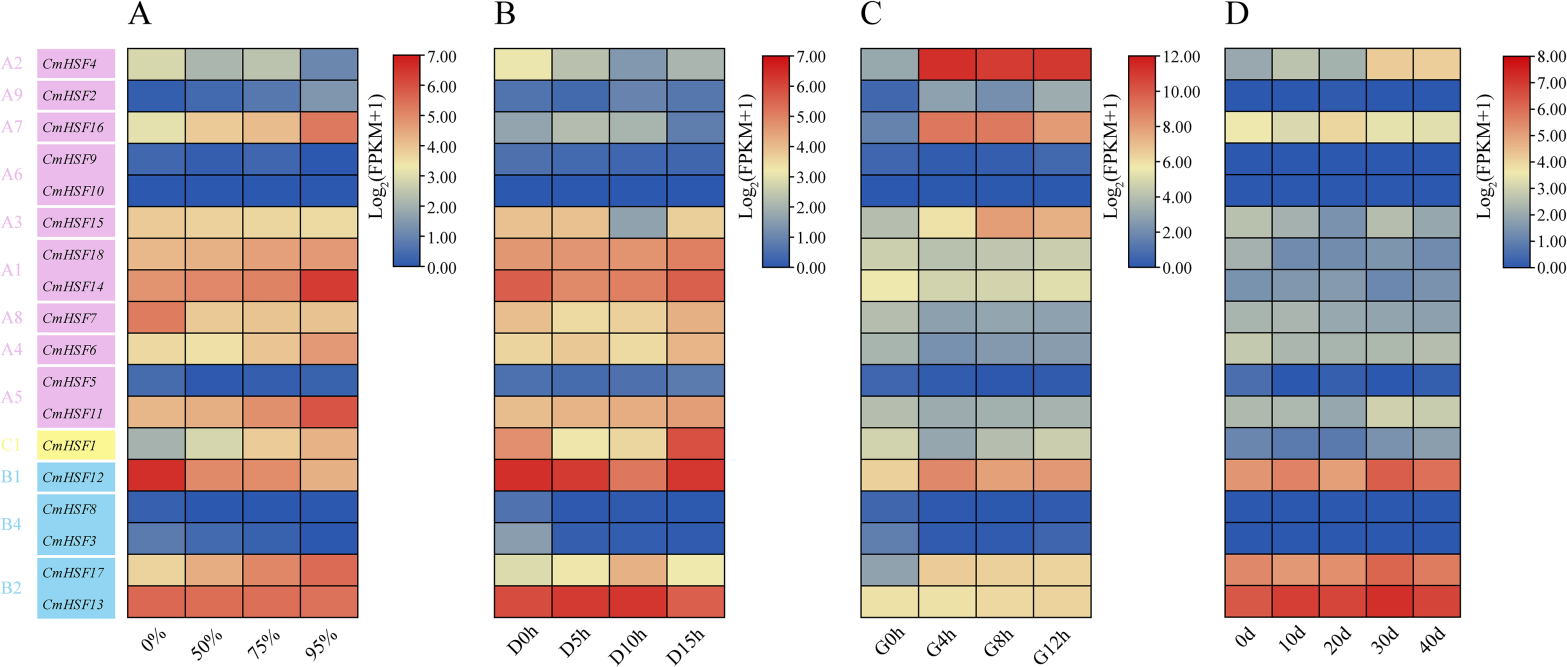
The expression pattern of *CmHSF* genes. **(A)** 0%, 50%, 75%, and 90% respectively represent different shading conditions. **(B)** D0h, D5h, D10h, D15h respectively represent the results after low-temperature treatment for 0 hours,5 hours, 10 hours, and 15 hours. **(C)** G0h,G4h, G8h, G12h respectively represent the results obtained after high-temperature treatment for 0 hours,4 hours, 8 hours, and 12 hours.The Roman numerals along the right-hand side of the figure indicate log_2_FPKM.

During the growth of a plant, it encounters various abiotic stresses that negatively affects its growth and development. To analyze the expression patterns of the *CmHSF* gene family under different abiotic stress conditions, a heat map was created based on the transcriptome data. The results showed that the expression levels of five genes (*CmHSF3*, *CmHSF5*, *CmHSF8*, *CmHSF9*, and *CmHSF10*) were nearly zero under different stress treatments. In contrast, the expression levels of the other 12 genes were relatively high under the different stress conditions, although their expression patterns differed. Under shading stress, the expression levels of six genes gradually decreased with an increase in the shading intensity, while the expression levels of seven genes gradually increased (**Figure 6A**). Under low-temperature stress, in addition to the above-mentioned five genes, the expression level of *CmHSF2* was very low and was not considered. With an increase in the low-temperature treatment time, the expression levels of two genes gradually decreased, three genes gradually increased, three genes initially increased and then decreased, and four genes initially decreased and then increased (**Figure 6B**). As the intensity and duration of high-temperature stress increased, the expression levels of three genes gradually decreased, six genes gradually increased, one gene first increased and then decreased, and three genes first decreased and then increased (**Figure 6C**). Under drought stress, in addition to the above five genes, the expression of *CmHSF2* was very low and was not considered (**Figure 6D**). With increasing drought duration, the expression levels of four genes gradually decreased, four genes gradually increased, two genes first increased and then decreased, and two genes first decreased and then increased. Notably, the expression of *CmHSF4* increased by 324 times at 4 h of high temperature treatment. This gene exhibited the most drastic changes, and the qRT-PCR results confirmed the correctness of the transcriptome data (**Supplementary Figure S1**).

### 3.7 Subcellular localization of *CmHSF4* and transgenic validation

**Figure 7:**
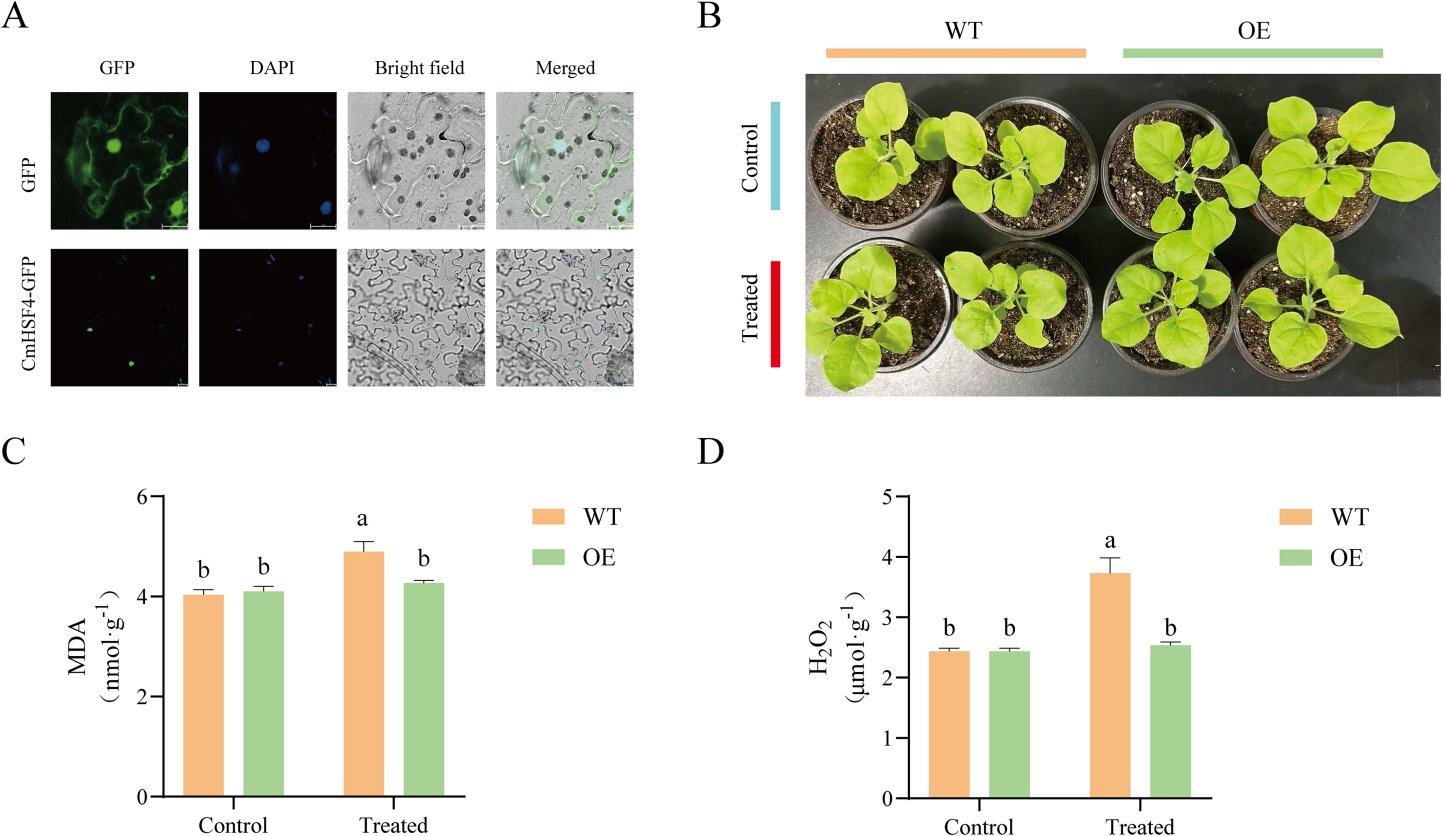
Functional characterization of the *CmHSF* gene. **(A)** Subcellular localization of *CmHSF4*. **(B)** The phenotype of tobacco after *CmHSF4* overexpression. **(C, D)** Determination of MDA and H_2_O_2_ contents in tobacco leaves.

To explore the subcellular localization of *CmHSF4*, we built a 35 s promoter CmHSF4–GFP fusion expression vector and converted *Arabidopsis* protoplasts. Confocal microscopy revealed that the GFP fluorescent signal exhibited a clear intracellular distribution pattern. More importantly, the GFP signal was completely colocalized with the staining region of the specific nuclear dye DAPI, confirming that *CmHSF4* is a nuclear protein (**Figure 7A**). This study also analyzed the effects of 42°C high-temperature stress for 5 min on tobacco seedlings (**Figure 7B**), by measuring the MDA and hydrogen peroxide (H_2_O_2_) contents of tobacco leaves. The results showed that after high-temperature treatment, the leaves of tobacco seedlings exhibited extensive water loss and chlorosis, with the leaves drying up and curling, and the contents of MDA and H_2_O_2_ showed an upward trend (**Figure 7C**), indicating that the tobacco seedlings suffered significant high-temperature damage following high-temperature stress.

## 4 Discussion

In the present study, we successfully classified 18 *CmHSF* members into three classes: A, B, and C. The distribution pattern of their numbers (class-A > class-B > class-C) was consistent with that of model plants, such as *Arabidopsis thaliana*, rice, and corn. Previous studies have also found that each type of *HSF* family in various plants is highly similar, and that the distribution of members is relatively similar. All were classified into three classes, A, B, and C, of which class A was the most common. This indicates that the *HSF* gene family has reached a certain degree of conservation in the plant kingdom. It is speculated that with the evolution of plants, members of the class-B and class-C may have lost some amino acids, whereas the amino acids between the bilateral heptavalent configuration (HR-A/B) composed of hydrophobic amino acid residues in members of the A *HSF* family are relatively complete, suggesting that members of the class-A *HSF* family may be more conserved than those of the B and C classes.

Phylogenetic analysis showed that multiple *CmHSF* members (such as *CmHSF12*/*13*/*17*) were clustered in the same evolutionary branch as the directly homologous genes in *Arabidopsis thaliana*, strongly suggesting that they may have similar functions. This inference was supported by gene structure analysis, which showed that members with close genetic relationships had similar motif compositions and gene structures (such as the number of introns/exons), whereas there were significant differences among different subfamilies, especially motif 6-10, which are specific to the Class-A subfamily and may determine its unique transcriptional regulatory function.

The expansion of gene families and functional differentiation are important driving forces for species adaptation to the environment. Collinearity analysis indicated that fragment replication was the key mechanism driving expansion of the *CmHSF* gene family. The Ka/Ks values of the two pairs of replicated genes were much lower than 1, indicating that this family has been subjected to intense purification selection pressure during evolution and that its protein sequences and functions have been highly conserved, which is crucial for maintaining its core biological functions. Further cross-species collinearity analysis provided clues for the functional differentiation of different subfamilies. Most Class-A members only had collinearity with dicotyledonous plants, suggesting that they might have emerged after the differentiation of monocotyledonous and dicotyledonous plants, and functionally might have been more specialized in the adaptability of woody dicotyledonous plants. Genes such as *CmHSF12*, *CmHSF13*, and *CmHSF17* were directly homologous in all analyzed species, including monocotyledonous plants, indicating that they are ancient and highly conserved members that may play an indispensable role in the core pathways of the plant kingdom in response to environmental stress.

Research on *CmHSF* upstream of the *cis*-elements of the 2000 bp sequence component revealed that the *CmHSF* promoter contains many light response elements, hormone response components, and adversity stress response elements. Considering that transcription factors can activate or inhibit transcription by binding to *cis*-elements in the promoters of related genes, it is speculated that some functional genes may regulate gene expression by binding to certain *cis*-elements in the *CmHSF* promoter, thereby participating in the regulation of adverse stress and affecting plant growth and development. However, related gene mining and specific regulatory methods require further investigation. In plants, *HSF* gene expression is mediated by abscisic acid (ABA) signaling. Upstream transcription factors can bind to ABRE to regulate the expression of *HSF* (Ohama et al., 2017), salicylic acid (SA), ethylene (Eth), and methyl jasmonate (MeJA), etc. stimulate the expression of the *HSF* gene in plants through hormone signaling pathways (Snyman) (Wang et al., 2022; Nie et al., 2022), thereby enhancing the tolerance of plants to high temperatures, indicating that plant hormones are widely involved in regulating the heat shock response (Clarke et al., 2009). Through our analysis of the *HSF* promoter region in Chinese chestnuts, numerous hormone response elements were identified, further indicating that plant hormones play a significant role in regulating the responses of plant *HSF* genes to heat shock. It is also known from the common cationic action elements of higher plant promoters, that the ABRE element is not only an abscisic acid signal response element, but also a high-temperature signal response element. In this study, ABRE elements were found in all 13 Chinese chestnut *HSF* genes, further indicating that Chinese chestnut *HSF* genes are widely involved in their responses to thermal shock.

This study also reports on the functions of some *HSF* transcription factors in the model plant *Arabidopsis thaliana*. By building a system for *Eucommia ulmoides* and members of the family of *Arabidopsis*, and according to the Chinese chestnut and *Arabidopsis thaliana* HSF clustering relationship, some gene functions can be predicted from the *HSF* evolutionary tree analysis; for example, *AtHsfA2* and *CmHSF4*. In this respect, *CmHSF4* has a relatively close genetic relationship and a similar evolutionary trend. Moreover, the over-expression of *AtHsfA2* in *Arabidopsis thaliana* enhances its tolerance to high-temperature stress, suggesting that *CmHSF4* may respond to high-temperature stress. An analysis of the expression patterns strongly verified the above prediction and revealed a diversity of functions among the family members. Under different abiotic stresses, *CmHSF* genes exhibit rich and complex expression profiles, indicating that although they belong to the same family, there may be a functional division of labor in stress responses. Notably, the expression level of *CmHSF4* was sharply upregulated (324 times) after 4 h of high-temperature stress. This phenomenon made it a crucial candidate gene for the response to heat stress in Chinese chestnuts. To verify its function, we conducted subcellular localization experiments and confirmed that *CmHSF4* was located in the nucleus, which is consistent with its expected function as a transcription factor. Subsequent functional verification experiments on transgenic tobacco demonstrated that high-temperature stress caused significant oxidative damage (increased MDA and H_2_O_2_ content) to tobacco seedlings; as such, further in-depth research should focus on the downstream target gene *CmHSF4* regulation to enhance heat tolerance in Chinese chestnuts.

Our findings, in conjunction with recent studies, collectively delineate the HSF-mediated heat stress response mechanism as a multi-level, dynamic and coordinated complex network. Firstly, the core function of HSF lies in directly activating the transcription of downstream protective genes. For instance, *MdHSFA2* in apples has been confirmed to directly bind to and activate the promoter of *MdGolS4*, thereby enhancing heat tolerance (Li et al., 2025). This study predicts that *CmHSF4* may have a similar function, and its nuclear localization feature is consistent with the role model of a transcription factor. Secondly, there is a fine functional division of labor and collaboration within the HSF family. Traditionally less studied C-class HSFs, such as *LlHSFC2* in lilies, have been proven to act as co-activators of transcription, significantly enhancing the transactivation ability of A-class HSFs (such as HSFA1, HSFA2, HSFA3) through heterologous interactions, and this interaction is specifically promoted after heat stress.

More importantly, the HSF pathway does not operate in isolation but extensively interacts with other signaling pathways, forming a larger regulatory network. Promoter analysis revealed that all *CmHSF* genes contain the abscisic acid (ABA) response element ABRE, which is not only a marker of ABA signaling but also a high-temperature response element, indicating that ABA signaling is deeply involved in the transcriptional regulation of Chinese chestnut *HSF* genes. This regulatory level may be very complex: on the one hand, transcription factors from other families can directly regulate *HSF* expression. For example, the HD-Zip I transcription factor *LlHB16* in lilies can directly bind to and activate the promoters of *LlHSFA2* and *LlMBF1C*, thereby positively regulating heat tolerance and linking the basic heat response pathway with ABA signaling. On the other hand, some transcription factors may also exert negative regulation on the HSF pathway. For instance, *LlERF110*, an AP2/ERF member in lilies, although induced by heat, its overexpression disrupts ROS homeostasis and inhibits the expression of heat protection genes (such as *AtHSFA2*), ultimately leading to reduced heat tolerance. Combining previous research, we speculate that *CmHSF4* may directly regulate downstream heat protection genes (such as *HSP*s or *Gol*S, etc.) through a mechanism similar to that of *MdHSFA2*; at the same time, it may also act as a network node, interacting with other members within the family (such as C-class HSFs) or transcription factors from other families to jointly integrate hormone signals such as ABA, precisely regulating the heat stress adaptability and growth and development balance of Chinese chestnuts. These findings lay a theoretical foundation for understanding the molecular mechanism of heat tolerance in woody plants and provide excellent candidate genes such as *CmHSF4* for molecular breeding of Chinese chestnut stress resistance. Future work should verify the downstream target genes of *CmHSF4* through EMSA and dual-luciferase reporter systems, and clarify its function at the plant level using transgenic technology, while further exploring its interaction with proteins such as CmHSFC to comprehensively map the transcriptional regulatory network of Chinese chestnut heat stress response.

## Data Availability

The original contributions presented in the study are included in the article/**Supplementary Material**. Further inquiries can be directed to the corresponding author.

## Supplementary material

Supplementary Figure 1 Expression analysis of *CmHSF4* under high temperature.G0h,G4h, G8h, G12h respectively represent the results obtained after high-temperature treatment for 0 hours,4 hours, 8 hours, and 12 hours.

Supplementary Table 1 The Ka, Ks, Ka/Ks Values of Gene Pairs Identified by McScanX.
