# Correction: Genome-wide identification of the *HSF* gene family in Chinese chestnut and functional characterization of *CmHSF4* under temperature stress

**DOI:** 10.3389/fpls.2026.1834026

**Published:** 2026-04-17

**Authors:** XiuRong Xu, ZiQi Wu, Xibing Jiang, Shiming Cheng

**Affiliations:** 1 Zhejiang Academy of Forestry, Hangzhou, Zhejiang, China; 2 Zhengzhou University, Henan, China; 3 Research Institute of Subtropical Forestry, Chinese Academy of Forestry, Hangzhou, China

**Keywords:** Chinese chestnut, gene family, genome-wide identification, HSF, temperature stress

Author “ZiQi Wu” was erroneously omitted as equal contributing author. “XiuRong Xu” and “ZiQi Wu” contributed equally to the work and share first authorship. The original version of this article has been updated.

