# Supplementary figures and images for: Genome-wide identification of the HSF gene family in Chinese chestnut and functional characterization of CmHSF4 under temperature stress
Source: Front Plant Sci. 2026 Mar 6;17:1749489. doi: 10.3389/fpls.2026.1749489 (PMC13002451; doi:10.3389/fpls.2026.1749489)

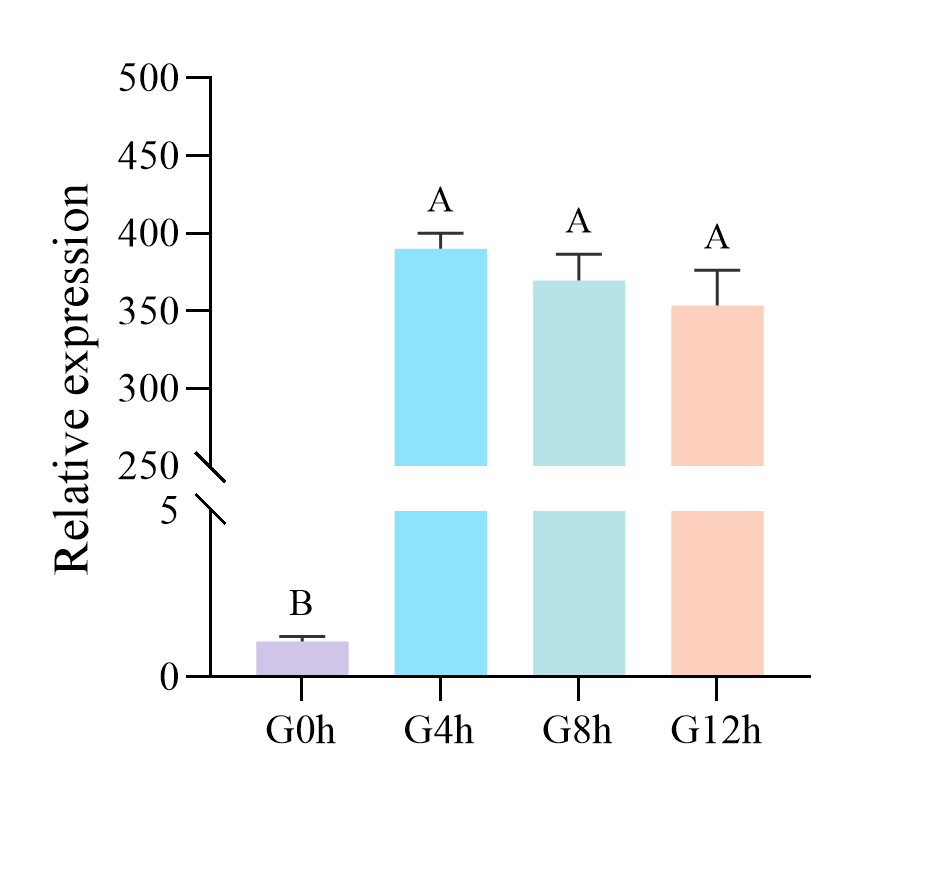

Supplement: Supplementary Figure 1 — Expression analysis of CmHSF4 under high temperature.G0h,G4h, G8h, G12h respectively represent the results obtained after high-temperature treatment for 0 hours,4 hours, 8 hours, and 12 hours. [file Image1.tif]
